# Sindbis viral vector induced apoptosis requires translational inhibition and signaling through Mcl-1 and Bak

**DOI:** 10.1186/1476-4598-9-37

**Published:** 2010-02-12

**Authors:** Lisa Venticinque, Daniel Meruelo

**Affiliations:** 1 NYU Cancer Institute and the NYU Gene Therapy Center, NYU School of Medicine, 550 First Avenue, New York, NY 10016, USA

## Abstract

**Background:**

Sindbis viral vectors are able to efficiently target and kill tumor cells *in vivo*, as shown using pancreatic and ovarian cancer models. Infection results in apoptosis both *in vitro *and *in vivo*. Sindbis vector uptake is mediated by the LAMR, which is upregulated on a number of different tumor types, thus conferring specificity of the vector to a wide range of cancers. In this study we elucidate the mechanism of apoptosis in two tumor cell lines, MOSEC, derived from the ovarian epithelium and Pan02, derived from a pancreatic adenocarcinoma. A comprehensive understanding of the mechanism of apoptosis would facilitate the design of more effective vectors for cancer therapy.

**Results:**

The initial phase of Sindbis vector induced apoptosis in MOSEC and Pan02 models reconfirms that viral infection is sensed by PKR due to double-stranded RNA intermediates associated with genomic replication. PKR activation results in translation inhibition through eIF2α phosphorylation and initiation of the stress response. Our studies indicate that the roles of two proteins, Mcl-1 and JNK, intimately link Sindbis induced translational arrest and cellular stress. Translational arrest inhibits the synthesis of anti-apoptotic Bcl-2 protein, Mcl-1. JNK activation triggers the release of Bad from 14-3-3, which ultimately results in apoptosis. These signals from translational arrest and cellular stress are propagated to the mitochondria where Bad and Bik bind to Bcl-xl and Mcl-1 respectively. Formation of these heterodimers displaces Bak, which results in caspase 9 cleavage and signaling through the mitochondrial pathway of apoptosis.

**Conclusion:**

The host cell response to Sindbis is triggered through PKR activation. Our studies demonstrate that PKR activation and subsequent translational arrest is linked to both cellular stress and apoptosis. We have also found the linkage point between translational arrest and apoptosis to be Mcl-1, a protein whose constant translation is required for inhibition of apoptosis. With this information vectors can be designed, which express or repress proteins implicated in this study, to enhance their therapeutic potential.

## Background

Current cancer therapies including chemotherapy, radiation and surgical resection remain inefficient at shrinking tumor burden and improving patient prognosis. While there have been improvements in the 5 year survival of patients diagnosed with ovarian cancer the cure rate remains at only 30% [1]. For pancreatic cancer the prognosis is even more grim; within one year of diagnosis 90% of patients succumb to cancer [2]. A targeted gene therapy approach could dramatically increase therapeutic efficacy and improve patient prognosis.

Sindbis virus is a positive single-stranded enveloped alphavirus from the *Togaviridae *family [3]. A replication defective vector derived from Sindbis virus has been used to treat tumors in mice [4-6]. Because of the blood-borne nature of this vector, it is delivered systemically and can therefore treat not only the primary tumor but sites of metastasis as well [4,5]. This vector is able to effectively target and efficiently shrink tumor burden from a number of xenograft models of cancer including pancreatic, colon [6] and ovarian cancers [4-7]. Sindbis vectors are also able to target spontaneous tumors shown in RGR/p15^+/- ^transgenic mice [5] and ovarian xenograft tumors implanted in immune competent mice [6]. These vectors have also been engineered to deliver genes, such as interleukin 12, which has enhanced the therapeutic potential [4]. The broad range of animal models in which Sindbis vectors have shown therapeutic efficacy, coupled with the ability to tailor therapies through the inclusion of a gene of interest, underscores the benefit of this vector for gene therapy.

Sindbis viral particles are able to attach to the surface of cells via the LAMR [8]. This characteristic enables the vector to target a wide range of tumor tissues in part due to the upregulation of LAMR on the surface of transformed cells [9-13]. After binding to LAMR the Sindbis viral particle is endocytosed and enters the endosomal system. Acidification in the endosomal compartment exposes E1, the fusogenic viral element, allowing fusion with the endosomal membrane, uncoating and entrance into the cytoplasm [3,14]. The RNA is then translated by the cellular machinery to form the replicase, comprised of four nonstructural proteins [3]. The replicase complex synthesizes the viral negative strand, which serves as the template for new copies of the viral genome and the shorter subgenomic RNA. Transcription of the negative strand templates to prepare positive strand genomic and subgenomic RNAs results in temporary double-stranded RNA products [15,16].

Infection with Sindbis virus causes a wide-scale cellular response resulting in significant changes in host cell physiology [17-23]. Previous *in vivo *studies using TUNEL staining confirmed that treatment of tumors with Sindbis vector induces apoptosis [6,24]. Therefore, a thorough understanding of the mechanism by which Sindbis vector induces apoptosis is crucial to developing more efficient viral vectors. Our study has extended and modified the prior understanding of the cellular response to Sindbis infection through systematic dissection of the apoptotic pathways. The double-stranded RNA intermediates, generated by Sindbis vector replication [15,16], are recognized by PKR [25]. PKR activation results in significant changes to the cell, which manifest as both cellular stress and translational inhibition through eIF2α phosphorylation [26,27]. Prior to this study the cellular stress response has not been studied in the context of Sindbis infection. Infection induces JNK phosphorylation, which plays a direct role in activating apoptosis. Sindbis infection also results in translational arrest, which not only inhibits new protein synthesis but also directly leads to apoptosis, as shown here.

Several studies have implicated different mechanisms by which Sindbis virus infection leads to apoptosis [17-22,28]. Apoptosis is triggered by one of two main mechanisms, the extrinsic, receptor-mediated pathway activated by members of the TNFα family of ligands and receptors [29]; or the intrinsic, mitochondrial pathway, which is triggered by intracellular signaling and involves members of the Bcl-2 family [30,31]. The Bcl-2 family is comprised of both pro-apoptotic proteins, (Bak and Bax), anti-apoptotic proteins, (Bcl-2, Bcl-xl and Mcl-1), and BH3 only proteins, (Bad, Bik and Noxa) which act as sensors [30,31]. Mitochondrial apoptosis proceeds when changes in subcellular localization or heterodimeric state render pro-apoptotic Bcl-2 proteins free to oligomerize, resulting in the release of cytochrome c and cleavage of caspase 9 [32,33]. By using targeted siRNA, we demonstrate that the mitochondrial pathway is the primary mechanism of Sindbis-induced apoptosis. The importance of several members of the Bcl-2 family, not previously implicated in Sindbis-induced apoptosis, is also illustrated.

Our results extend and clarify previous studies in the literature and provide a better understanding of Sindbis vector-host cell interactions. Comprehensive study of the cellular response, focusing on changes in translation and apoptosis, will enable the production of a more efficient Sindbis viral vector for gene therapy.

## Results

In these studies we have chosen to use MOSEC, a murine epithelial ovarian cancer cell line, because these cells have been used previously in a xenograft mouse model for *in vivo *treatment with Sindbis vector [4,6]. The murine pancreatic adenocarcinoma Pan02 cells have also been used to confirm critical results, as an additional cancer model of different tissue origin, verifying that the results shown are not restricted to a single tumor cell line or tissue type. These cells have also been used previously as an *in vivo *cancer model [5].

### PKR senses viral dsRNA species

**Figure 1:**
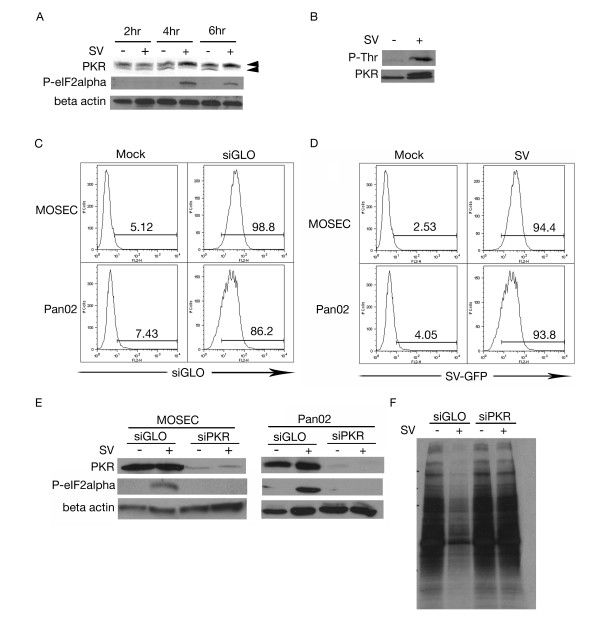
**dsRNA species activate PKR**. (A) Sindbis vector (SV) infection leads to activation of PKR. Lysates collected at 2, 4 and 6 h.p.i. from mock (-) or SV infected (+) MOSEC cells were analyzed by immunoblotting using a polyclonal antibody for PKR (upper arrow represents the phosphorylated form, lower arrow, non-phosphorylated form). (B) PKR is threonine phosphorylated following infection. Lysates were subjected to immunoprecipitation with PKR and blotted for phospho-threonine. SV infected MOSEC cells but not mock infected cells show phosphorylation indicating that this event is the result of infection. (C) MOSEC and Pan02 cells are efficiently transfected with siRNA. MOSEC and Pan02 cells were transfected with siGLO, a fluorescently conjugated control siRNA. Cells were collected and subjected to FACS analysis. (D) SV-GFP efficiently infects both MOSEC and Pan02 cells. 16 h.p.i. with SV-GFP, cells were harvested for FACS analysis monitoring GFP expression. (E) eIF2α phosphorylation is inhibited in cells with diminished PKR expression. Lysates were collected from MOSEC or Pan02 cells, transfected with siPKR or siGLO and infected with SV or mock infected, at 6 h.p.i. Immunoblotting for phospho-eIF2α indicates a lack of phosphorylation in siPKR-transfected cells. Immunoblots in panels A and E were stripped and reprobed with β actin for loading control. (F) Transfection of siPKR inhibits Sindbis-induced translational arrest. MOSEC cells transfected with either siPKR or siGLO were infected with SV or mock infected and subjected to ^35^S labeling. Decreased translation was observed in the siGLO transfected/SV infected sample however not the similarly treated siPKR sample.

In mammalian cells PKR acts as a sensor of double-stranded RNA and can promote a potent antiviral response upon activation [34]. Due to the double-stranded RNA intermediates generated by Sindbis vector replication, we studied the effect of Sindbis infection on PKR. We infected MOSEC cells with Sindbis vector and examined cell lysates at 2, 4 and 6 h.p.i Western blot analysis showed an upward mobility shift in PKR between 2 and 4 h.p.i. indicating that phosphorylation had occurred (Figure 1A).

Immunoprecipitation of PKR and blotting for phospho-threonine confirmed that the mobility shift was the result of a phosphorylation event (Figure 1B). The main downstream target of PKR is eIF2α. Western blot analysis indicates that Sindbis vector infection induces eIF2α phosphorylation (Figure 1A) indicating translational inhibition.

To characterize the downstream effects of PKR activation in response to Sindbis vector, siPKR was employed. Use of siGLO, a fluorescently labeled siRNA, enabled the calculation of transfection efficiency (Figure 1C). Infectivity was also monitored by FACS analysis (Figure 1D). Western blotting of siPKR transfected cells, infected with Sindbis vector, indicates the expected lack of eIF2α phosphorylation (Figure 1E). Downstream translational arrest was assessed through ^35^S- methionine labeling of siPKR-transfected cells (Figure 1F). The results implicate PKR as the cellular sensor of Sindbis infection.

**Figure 2:**
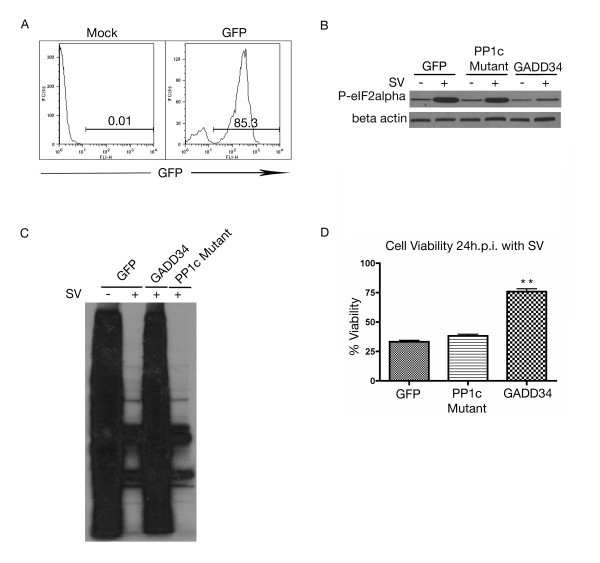
**Translational arrest is inhibited by ablation of PKR expression**. (A) MOSEC cells are efficiently transfected with GFP control vector. At 48 hours post transfection MOSEC cell were harvested for FACS analysis monitoring FL-1 for GFP expression. (B) Cells transfected with GADD34, but not the GADD34 PP1c deletion mutant, can dephosphorylate eIF2α. MOSEC cells transfected with GADD34, PP1c mutant or GFP control vector were infected at 48 hours. 8 h.p.i. lysates were collected and subjected to western blotting for phospho-eIF2α. In panel B immunoblot was stripped and reprobed with β actin for loading control. (C) Transfection of GADD34 but not the PP1c mutant alleviates Sindbis vector-induced translational arrest. MOSEC cells transfected with GADD34, PP1c mutant or GFP control vector were infected at 48 hours. 24 h.p.i. cells were subjected to ^35^S labeling. (D) Dephosphorylation of eIF2α by GADD34 but not the GADD34 mutant lacking the PP1c interacting domain, increases the cell viability at 24 h.p.i. MOSEC cells were transfected with either GADD34, GADD34 PP1c mutant or GFP control vector. 48 hours after transfection cells were infected with SV. 24 h.p.i. cell viability was assessed. Data in D represents the SEM (error bars) of three experiments. Cell viability for each sample was compared to the infected control at the same time point and was corrected for the percentage of infected cells. Statistical significance was calculated by a two-tailed student t-test (** P < 0.005).

GADD34 promotes recovery from translational arrest by binding PP1c and inducing dephosphorylation of eIF2α [35]. To confirm the importance of eIF2α in Sindbis vector infection, MOSEC cells were transiently transfected with GADD34 or a mutant form lacking the PP1c interacting domain. Transfection of a control vector expressing GFP, enabled the calculation of transfection efficiency (Figure 2A). Western blotting for phospho-eIF2α indicated that GADD34, but not the PP1c mutant or GFP control, was able to dephosphorylate eIF2α (Figure 2B). Overexpression of GADD34 was able to alleviate the inhibition of translation caused by Sindbis infection (Figure 2C) and also significantly increase cell viability (Figure 2D). This data indicates that translational arrest is an essential step in the cellular response leading to apoptosis.

### Sindbis vector infection activates the cellular stress response

**Figure 3:**
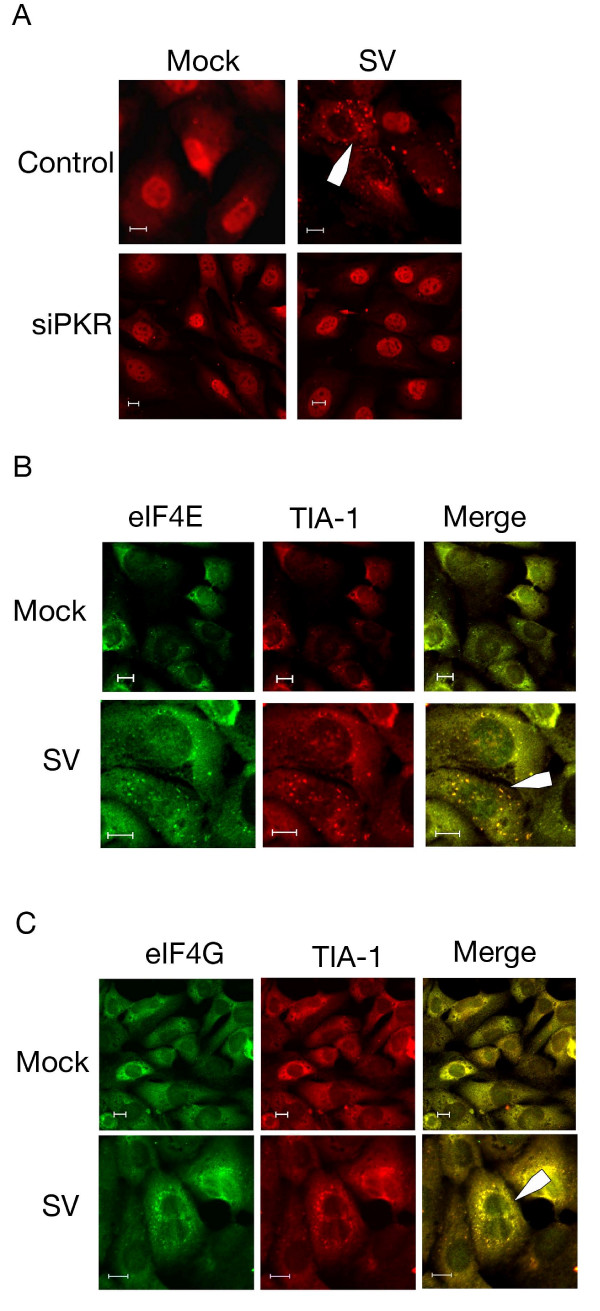
**Sindbis vector infection induces the formation of stress granules**. (A) Infection with Sindbis vector induces the formation of stress granules, which can be inhibited by siPKR transfection. MOSEC cells transfected with siPKR or siGLO and infected with SV or mock infected were processed for immunofluorescence for stress granule marker TIA-1 at 6 h.p.i. Arrows in each panel indicate stress granules. (B&C) Sindbis vector infection results in the sequestration of translation initiation factors in stress granules. MOSEC cells were infected with SV or mock infected and processed for immunofluorescence using antibodies for TIA-1 and eIF4E (B) or eIF4G (C) at 6 h.p.i. Co-localization of translation initiation factors with TIA-1 indicates that they are located in stress granules following infection. Scale bars for panels A-C indicate 10 μm.

Viral infection often activates pathways associated with cellular stress and can manifest in the formation of stress granules. These granules are dynamic cytoplasmic structures used to sequester cellular RNA and translational machinery until normal translation can be restored [15,16]. To determine if stress granules form in response to Sindbis infection, cells were stained for TIA-1, an RNA binding protein, which serves as a marker for these structures. TIA-1 can be localized to the nucleus or cytoplasm in untreated cells as it shuttles between both subcellular localizations [36]. In infected cells, immunofluorescence revealed the appearance of punctate structures localized within the cytoplasm at 6 h.p.i. (Figure 3A). The lower panels of Figure 3A indicated that these structures do not form in cells where PKR expression has been knocked down by siRNA. Therefore, Sindbis-induced stress granule formation is contingent upon PKR activation.

To characterize the content of the stress granules, immunofluorescence was performed using antibodies that recognize different components of the cellular translation initiation machinery. Following infection with Sindbis vector, both eIF4E (Figure 3B) and eIF4G (Figure 3C) are contained within punctate structures and co-localize with TIA-1 indicating that they are located in stress granules. The presence of translation initiation machinery likely indicates a secondary mechanism of translational inhibition.

**Figure 4:**
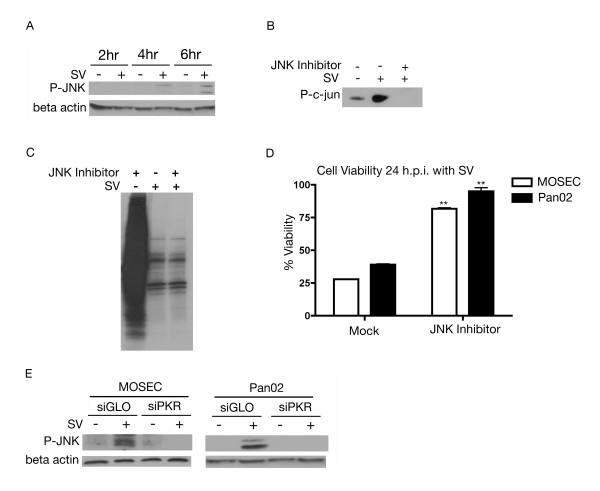
**Sindbis vector infection activates JNK**. (A) JNK phosphorylation is induced by infection with Sindbis vector. Lysates of SV (+) infected or mock (-) infected MOSEC cells were collected at 2, 4 and 6 h.p.i. Immunoblotting with phospho-JNK first detects phosphorylation at 4 h.p.i. (B) Cell permeable JNK inhibitory peptide inhibits c-jun phosphorylation. MOSEC cells were treated for 1 hour with JNK specific inhibitory peptide and then infected with SV or mock infected and subjected to a JNK specific kinase assay 24 h.p.i. (C) Inhibition of JNK activation has no effect on Sindbis-induced translational arrest. MOSEC cells treated with JNK inhibitor and infected were labeled with ^35^S methionine 24 h.p.i. (D) JNK inhibition results in an increase in cell viability following Sindbis infection. MOSEC or Pan02 cells pretreated for 1 hour with a JNK-specific peptide inhibitor and then infected with SV or mock infected were assessed for cell viability 24 h.p.i. Data in D represents the SEM (error bars) of three experiments. Each sample was compared to the non-treated infected control at the same time point. Statistical significance was calculated by a two-tailed student t-test (** P < 0.005). (E) JNK phosphorylation is downstream of PKR activation. Lysates from MOSEC or Pan02 cells, transfected with siPKR or siGLO (control) and infected with SV or mock infected, were collected at 8 h.p.i. Immunoblotting for phosphorylated JNK indicates that it remains dephosphorylated in cells treated with siPKR. The immunoblots in A&E were stripped and reprobed for β actin as a loading control.

A major part of the cellular stress response involves stress kinases, which are able to propagate the stress signal from the detection phase and evoke a cellular response [26,27,37]. Activated PKR can mediate JNK activation [26,38], whose signaling pathway mediates processes such as cell proliferation and apoptosis [37]. We assessed the role of JNK in Sindbis infection. At 4 h.p.i. JNK was phosphorylated and therefore activated (Figure 4A). To confirm that JNK activation is the result of Sindbis infection, MOSEC cells were treated with a cell-permeable JNK-specific peptide inhibitor. A JNK-specific kinase assay followed by western blotting for phospho-c-jun confirmed inhibition of JNK (Figure 4B). Therefore, Sindbis infection induces JNK activation.

As shown in Figure 1 Sindbis vector infection induces translational shut off. To elucidate the role of activated JNK in this phenomenon, cells were subjected to ^35^S labeling after treatment with JNK inhibitor and infection. Reduction in translation was observed 24 h.p.i. in the presence or absence of JNK inhibitor, indicating that JNK activation had no effect on Sindbis-induced translational arrest. No changes were observed in JNK inhibited, mock-infected cells, which excludes any effect of the JNK inhibitor on translational arrest (Figure 4C).

JNK activation is capable of inducing apoptosis through downstream activation of transcription factors and phosphorylation of target proteins [37]. MOSEC or Pan02 cells were treated with an inhibitory peptide and infected. We found that JNK activation is related to a loss of cell viability in Sindbis infected cells. With inhibition of JNK, infected cells remain nearly 100% viable 24 h.p.i. (Figure 4D). This result is common to both ovarian and pancreatic cell lines and underscores the importance of JNK activation and cellular stress in the host cell response.

To assess the importance of PKR in stress kinase activation, the phosphorylation status of JNK was studied in cells where the expression of PKR was attenuated. In these cells JNK remains dephosphorylated (Figure 4E). This result was observed in both MOSEC and Pan02 cell lines. The lack of JNK phosphorylation in PKR knockdown cells indicates that JNK activation is contingent upon PKR activation.

### Initiation of the apoptotic response

**Figure 5:**
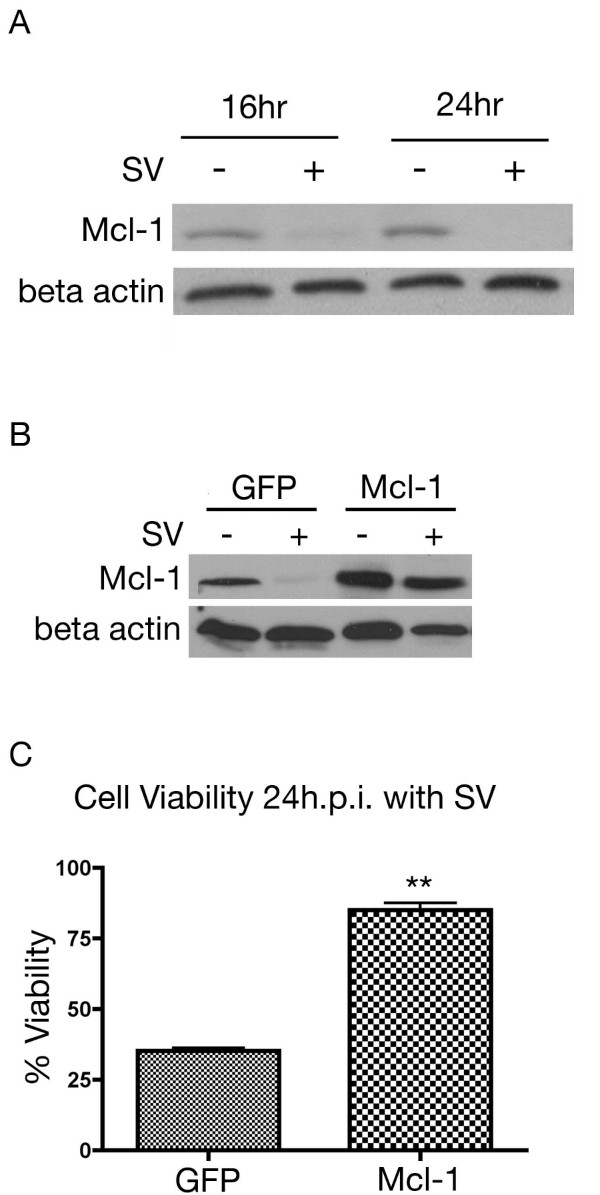
**Role of Mcl-1 in Sindbis infection**. (A) Sindbis vector infection induces the degradation of Mcl-1. Lysates were collected from MOSEC cells infected with SV (+) or mock (-) infected. Immunoblot analysis indicates a loss of Mcl-1 expression by 16 h.p.i. (B) MOSEC cells transfected with Mcl-1 vector overexpress the protein. Cells transfected with Mcl-1 or GFP control vector were infected at 48 hours post transfection. At 24 h.p.i. lysates were collected and subjected to western blotting for Mcl-1. In A and B immunoblots were stripped and reprobed with β actin for loading control. (C) Mcl-1 overexpression protects the cells from Sindbis-induced loss of viability. MOSEC cells were transfected with Mcl-1 or GFP transfected. At 48 hours cells were infected with Sindbis vector. 24 h.p.i. cell viability was assessed. Data in C represents the SEM (error bars) of three experiments. Each sample was compared to the infected control at the same time point. Statistical significance was calculated by a two-tailed student t-test (** P < 0.005).

The Mcl-1 protein is rapidly turned over in normal cells. In cells with a reduced translational capacity due to nutrient deprivation, stress or viral infection, Mcl-1 protein levels are markedly reduced. Without Mcl-1 to bind and sequester Bak, the cell becomes susceptible to apoptosis [39]. Through western blotting we observed a loss of Mcl-1 protein, 16 h.p.i. (Figure 5A). Overexpression of Mcl-1, confirmed by western blotting (Figure 5B), was able to rescue cell viability 24 h.p.i. (Figure 5C). The ability of Mcl-1 overexpression to protect cell viability indicates that loss of this protein due to translational arrest is important to the downstream apoptotic response.

We have shown that JNK is activated as part of the cellular stress response to Sindbis infection (Figure 4). Activated JNK has been linked to apoptosis through disruption of the complex between 14-3-3 and Bad, enabling Bad to translocate to the mitochondria [40,41]. Immunoprecipitation of cytoplasmic and mitochondrial cell fractions with antibodies to Bcl-2 family proteins reveals this process in Sindbis vector infected cells.

**Figure 6:**
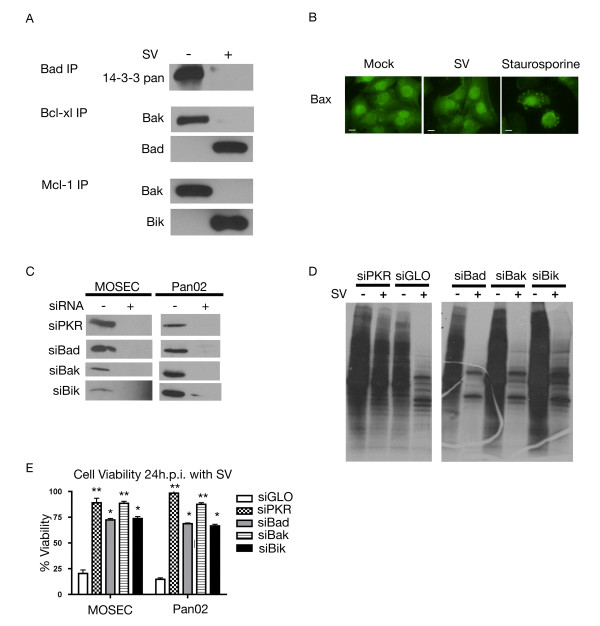
**Involvement of Bcl-2 proteins in the cellular response**. (A) Infection with Sindbis results in a shift in anti-apoptotic Bcl-2 family heterodimer composition. Cell lysates were collected from SV (+) or mock (-) infected MOSEC cells 20 h.p.i. and subjected to mitochondrial isolation. Fractionated lysates were immunoprecipitated with antibodies specific to Bad (cytoplasmic fraction), Bcl-xl and Mcl-1 (mitochondrial fraction). (B) Bax remains in the cytoplasm following infection with Sindbis vector. MOSEC cells were infected with SV-Luc or treated with staurosporine, as positive control. At 20 h.p.i. samples were subjected to immunofluorescence staining for Bax. Scale bars indicate 10 μm. (C) Knockdown of proteins using targeted siRNA is significant. MOSEC or Pan02 cells were transfected with indicated siRNAs. Lysates were collected 24 h.p.i. and subjected to western blotting for indicated proteins. (D) Translation is still inhibited in cells after protein knockdown. MOSEC cells transfected with siRNA directed against indicated proteins and infected with SV or mock infected were subjected to ^35^S methionine labeling 24 h.p.i. (E) Ablation of expression of certain BH3 only proteins can partially protect cells from Sindbis vector infection. MOSEC or Pan02 cells were treated with indicated siRNAs (or siGLO control) and infected with Sindbis vector. Cell viability was assessed 24 h.p.i. Data in E represents the SEM (error bars) of three experiments. Cell viability for each sample was compared to the infected control and corrected for percentage of infection. Statistical significance was calculated by a two-tailed student t-test (* P < 0.05, ** P < 0.005).

Following Sindbis infection, immunoprecipitation of the cytoplasmic fraction of MOSEC cells with Bad antibody indicates that 14-3-3 is released from this complex (Figure 6A). Moreover, through immunoprecipitation of the mitochondrial fraction with Bcl-xl antibody, we confirmed that Bad did translocate to the mitochondria and that it binds to Bcl-xl (Figure 6A). We also observed that Bak, which binds to Bcl-xl in the mitochondrial fraction of uninfected cells is released from this complex after infection (Figure 6A). The shift in heterodimeric species within the mitochondria illustrates how the apoptotic signal is translated from the cytoplasm.

Signaling through the mitochondrial apoptotic pathway proceeds when either Bax translocates to the mitochondria or when dimers consisting of Bak and anti-apoptotic proteins are disrupted [31,42,43]. Following infection with Sindbis vector we did not observe a change in Bax cellular distribution. In untreated (mock) cells Bax is located in the cytoplasm (Figure 6B), Bax remains in the cytoplasm following Sindbis infection (Figure 6B), indicating that it is not a key player in the cellular response.

To isolate the key members of the Bcl-2 family, directed siRNA against Bad, Bak and Bik was employed. siPKR transfected samples were used as a control. Successful knockdown was confirmed by western blotting (Figure 6C) and ^35^S labeling confirmed that translational arrest is not affected by knockdown (Figure 6D). Attenuation of Bak expression induced a dramatic increase in cell viability (Figure 6E), indicating a reduction in apoptosis and underscoring the importance of the mitochondrial pathway.

It has been described that Bak can be displaced from its complex with Mcl-1 by either Bik or Noxa [42,44]. To determine the importance of Bik in the cellular response, siRNA was used to ablate its expression. Knockdown of Bik resulted in an increase in cell viability (Figure 6E). The modest change in cell viability most likely results from redundancy within the BH3 only proteins. We then investigated the effect of Sindbis infection on the heterodimeric species of Mcl-1. Through the use of mitochondrial isolation followed by immunoprecipitation with Mcl-1 antibody we were able to study the heterodimeric species of the modest amount of Mcl-1 protein remaining in the cell. Following infection, immunoprecipitation indicated that Bik was bound to Mcl-1. We also found that Bak was absent from this complex following infection. (Figure 6A). This validates the significance of Bik in the cellular response through its interaction with Mcl-1 and its role in downstream apoptosis.

It has been suggested that efficient activation of Bak requires its release from complex with both Bcl-xl and Mcl-1 [42]. Immunoprecipitation results in Figure 6A showed that upon Sindbis infection, Bad displaced Bak in its complex with Bcl-xl. siRNA was employed to study the role of Bad in Sindbis-induced apoptosis. In the absence of Bad expression, cells remain 75% viable after infection, indicating that Bad plays a role in inducing apoptosis, and acts in a manner similar to that of Bik (Figure 6E).

### Activation of the apoptotic cascade

**Figure 7:**
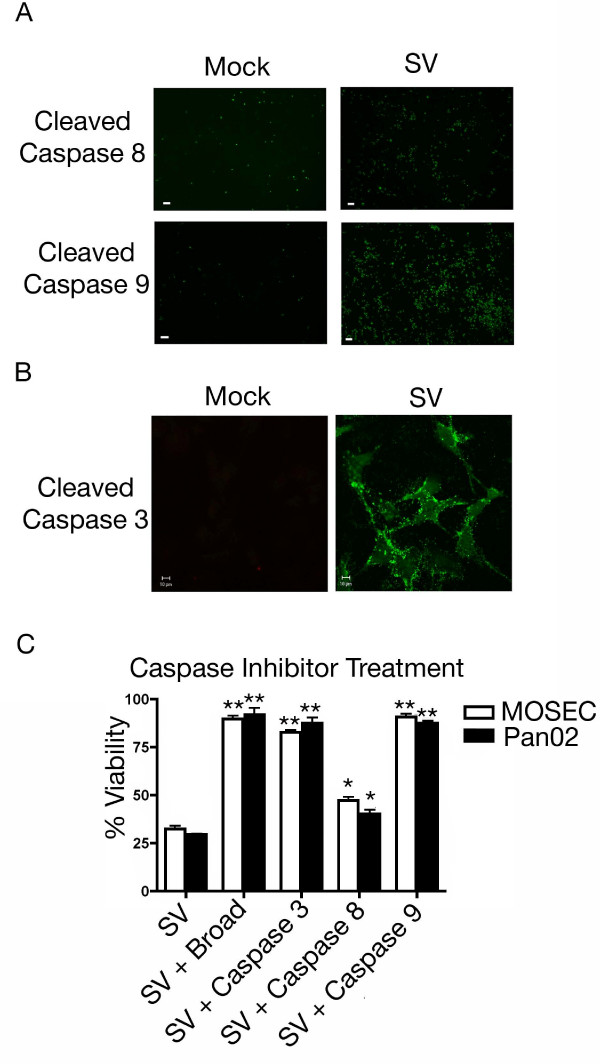
**Sindbis vector infection causes caspase cleavage**. (A) Sindbis vector infection leads to significant cleavage of caspase 9 but minimal amounts of caspase 8. MOSEC cells infected with SV or mock infected were assayed 24 h.p.i. for caspase activation using fluorescent probes specific for individual caspase cleavage products. Scale bars for panel A indicate 100 μm. (B) Infection with Sindbis viral vector results in the cleavage of caspase 3. MOSEC cells infected with SV or mock infected were assayed at 24 h.p.i. for caspase 3 cleavage using a cleavage specific fluorescent probe. Scale bars indicate 10 μm. (C) Treatment with selective caspase inhibitors can protect cells from a loss of cell viability. MOSEC or Pan02 cells were infected with Sindbis vector or mock infected and then incubated with caspase specific peptide inhibitors. Cell viability was assessed at 24 h.p.i. Cell viability indicates that cells treated with a broad caspase inhibitor, caspase 3 inhibitor or caspase 9 inhibitor are protected from apoptosis whereas samples treated with caspase 8 inhibitor were not. Data in C represents the SEM (error bars) of three experiments. Each sample was compared to the infected control at the same time point. Statistical significance was calculated by a two-tailed student t-test (* P < 0.05, ** P < 0.005).

Once there is involvement of the Bcl-2 family of proteins, the apoptotic cascade proceeds by a process that is relatively conserved [32,33]. Bak activation leads to a loss of mitochondrial membrane potential and cleavage of caspase 9 [45,46]. Cleavage of caspase 3 results in the activation of a large number of molecules able to complete the apoptotic process [46]. Immunofluorescence using a fluorescently labeled cell permeable probe with the ability to bind to activated caspases was employed. Infection resulted in the cleavage of a significant amount of caspase 9 however only a modest amount of caspase 8 (Figure 7A). Immunofluorescence also indicated the cleavage of caspase 3 following infection (Figure 7B). To study the roles of caspase 8 (receptor-mediated pathway), caspase 9 (mitochondrial pathway) and caspase 3 (effector caspase) in Sindbis-induced apoptosis, inhibitory peptides were used that bind to and irreversibly inhibit the active forms of each. As expected, cells treated with a broad caspase inhibitor remained nearly 100% viable (Figure 7C). This indicates that caspases are essential for Sindbis vector-induced apoptosis. When cells were treated with a caspase 8 inhibitor, there was still a significant loss in cell viability, only a modest change from Sindbis vector infection alone (Figure 7C), correlating with the modest caspase 8 cleavage seen in Figure 7A. Interestingly, when cells were treated with caspase 9 inhibitor, nearly 100% viability was maintained (Figure 7C). Downstream of caspase 9 is effector caspase 3 and, as expected, when cells were treated with an inhibitory peptide directed against caspase 3 there was little loss in cell viability (Figure 7C). These findings indicate that Sindbis vector-induced apoptosis in both ovarian and pancreatic tumor cell lines requires caspase 9 activation and proceeds through caspase 3.

## Discussion

In these studies we have focused on characterizing the cellular response to Sindbis infection using MOSEC and Pan02 cells. The use of tumor cell lines enabled us to evaluate the behavior of Sindbis vector in the type of cell where it would be used as a therapeutic. Key experiments were also performed in NIH3T3 cells in parallel as confirmation that the responses shown were not an artifact of using transformed cells (data not shown).

Previous investigations of the cellular response to Sindbis infection indicate that PKR is activated by the double-stranded RNA species generated by viral replication and that cellular translation is reduced [25]. We observed that once PKR is activated, a wide-scale cellular signaling process commences. However, this response extends beyond just translation inhibition. We have demonstrated that PKR activation induces both cellular stress and apoptosis. Both MOSEC and Pan02 cells have an intact type I IFN response, however the kinetics of IFN production and secretion do not account for the cellular effects occurring downstream of PKR activation (data not shown).

Our work using siRNA directed against PKR confirmed the importance of PKR in the cellular response and verified that it was responsible for eIF2α phosphorylation. In our tumor cell model, we confirmed that PKR is activated in response to Sindbis vector infection and also found that it is responsible for orchestrating the downstream cellular response.

The work of Ventoso et al. (2006) demonstrated that PKR is activated and subsequently inhibits translation, [25] however, its effect on the later cellular response and apoptosis was not explored. Translational arrest-induced stress manifests as stress granules, cytoplasmic foci which are aggregates of RNA binding proteins, as well as cellular mRNAs [15,16,47,48]. Previous work with SV and SFV, a related alphavirus, has shown the formation of stress granules [49,50]. By identifying the presence of translation initiation components within stress granules following infection we demonstrate the existence of a secondary mechanism used to inhibit translation in these cells. We extend our studies of the stress response to show that JNK is both activated and vitally important to apoptosis as evidenced by an increase in cell viability in Sindbis infected cells after treatment with a JNK-specific inhibitor.

Numerous studies have resulted in conflicting data concerning the mechanism by which Sindbis virus initiates apoptosis. Some studies have implicated viral binding alone as sufficient to activate the apoptotic cascade [20,21]. Our data, in MOSEC and Pan02 cells, indicates that binding alone is not sufficient to induce apoptosis but, rather, that viral replication is required since PKR activation is dependent on the presence of viral replication intermediates.

The mitochondrial apoptotic pathway is triggered by an intracellular mechanism that involves members of the Bcl-2 family. Through the use of directed siRNA, we establish that the mitochondrial pathway is the primary pathway to apoptosis. In our system we have demonstrated that Bad ablation is able to partially inhibit apoptosis. We also demonstrate that Bik, another BH3 only protein, not previously studied in the context of Sindbis-induced apoptosis, plays a similar role.

Our studies do not show an involvement of Bcl-2, but rather of Bcl-xl, a binding partner of Bak. Both proteins are anti-apoptotic members of the Bcl-2 family and therefore perform redundant functions having different binding/activating partners. Other studies of Sindbis-induced apoptosis have implicated different components of the Bcl-2 family. Several studies showed that overexpression of Bcl-2, was sufficient to inhibit apoptosis [18,19,22,51,52], one using Sindbis vector itself to overexpress Bcl-2 [51], and another implicated Bad [28]. It is possible that overexpression of Bcl-2 protein in these studies was able to generate a sufficient amount of protein to exert its normal cellular function and also inhibit Bak oligomerization. Our studies using Sindbis vector in tumor cell lines found that Bcl-xl is responsible for Bak release and that activation of apoptosis occurs through the mitochondrial pathway.

Activation of Bak requires release not only from its complex with Bcl-xl but also from Mcl-1 [42]. The mechanism of Mcl-1 regulation requires rapid turnover, therefore, in the infected cell with its reduced translational capacity, there is a loss of Mcl-1 expression and, consequently, its protective effect. Activation of apoptosis by Mcl-1 depletion has not been applied in the context of Sindbis virus before, and it is also a mechanism divergent from that seen in SFV infection [53]. This underscores the significance of PKR activation as a sensor of vector infection, as well as providing a crucial link between translational arrest and apoptosis.

Activation of caspase 8 has been implicated as a primary mechanism of Sindbis induced apoptosis [17]. Our data, using caspase 8-specific inhibitors indicates that, in infected tumor cell lines, this is a secondary mechanism, most likely activated in a feedback loop to enhance the apoptotic response. We have shown that caspase 9 inhibitory peptides protect cells from Sindbis-induced apoptosis suggesting that caspase 9 activation is the primary mechanism initiating the caspase cascade and leading to apoptosis.

## Conclusions

**Figure 8:**
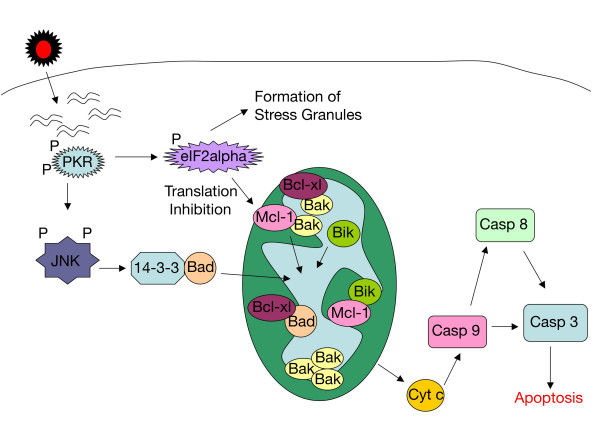
**Schematic diagram of the cellular response to Sindbis vector infection**. Sindbis vector replication forms double-stranded RNA species, which activate PKR. PKR is able to inhibit cellular translation through eIF2α phosphorylation and the formation of stress granules. Translation inhibition blocks the production of new Mcl-1, which is rapidly turned over. The remaining Mcl-1, in complex with Bak, is displaced by BH3 only protein Bik, thus releasing Bak. Downstream of PKR, JNK is activated, which causes the release of Bad from its complex with 14-3-3. Bad translocates to the mitochondria and disrupts the complex between Bcl-xl and Bak. Bak oligomerizes resulting in the release of cytochrome c from the mitochondria. Caspase 9 is cleaved causing caspase 3 cleavage and downstream apoptosis.

Figure 8 depicts the model of Sindbis vector-induced apoptosis that we have generated from our work in two distinct tumor cell lines. Replication of Sindbis viral vector is sensed by PKR, which results in the formation of stress granules and global translation inhibition through eIF2α phosphorylation. Translation inhibition also prevents the synthesis of new Mcl-1. PKR activates JNK, which phosphorylates 14-3-3 and disrupts its complex with Bad. Once Bad is released from 14-3-3 it translocates into the mitochondria. Both Bad and Bik displace Bak from its complexes with Bcl-xl and Mcl-1, respectively, and allow it to oligomerize. This step enables permeabilization of the mitochondrial membrane and the release of cytochrome c. The apoptosome forms in the cytoplasm and cleaves caspase 9, which in turn cleaves caspase 3 and activates downstream apoptotic signaling.

This study increases our knowledge of the Sindbis vector-host cell interactions that lead to apoptosis. With this new understanding of Sindbis-induced apoptosis, better vectors may be designed to eradicate tumor cells. Such vectors could be engineered to carry proteins that enhance the apoptotic response for more effective treatment of cancer.

## Methods

### Cell Lines

BHK cells were obtained from the American Type Culture Collection. Cells were maintained in culture in αMEM with 10% FCS. MOSEC, murine ovarian cancer cells were derived from the ovarian epithelium of C57BL6 mice, a generous gift from Dr. Terranova [54]. Cells were maintained in culture in DMEM 1 g/l glucose with 4% FCS and insulin (5 μg/ml), transferrin (5 μg/ml), and selenium (5 ng/ml). Pan02 cells, a murine pancreatic adenocarcinoma cell line with ductal morphology was obtained from the NCI-Frederick Cancer Research Facility. Cells were maintained in DMEM 1 g/l glucose with 10% FCS. All basal media was supplemented with 100 ug/ml of penicillin-streptomycin and 0.5 ug/ml amphotericin B (all from Mediatech).

### Inhibition Assays

JNK phosphorylation was inhibited through the use of cell permeable JNK inhibitory peptide VII (Calbiochem). Briefly, cells were incubated with JNK inhibitor 10 ug/ml in media for 1 hour at 37°C. Cells were infected as described below. Following infection, cells were incubated with JNK inhibitor for 24 hours. Cell viability was determined as described below. To assess the level of JNK inhibition, lysates were collected and subjected to the SAPK/JNK kinase assay kit, according to manufacturer instructions (Cell Signaling).

The activity of caspases was inhibited through the use of cell permeable inhibitors (BioVision). Briefly, cells were infected with SV-GFP or mock infected as described below. After infection, cells were incubated in media containing either broad caspase inhibitor Z-VAD-FMK, caspase 3 inhibitor Z-DEVD-FMK, caspase 8 inhibitor Z-IETD-FMK or caspase 9 inhibitor Z-LEHD-FMK at a concentration of 4 μM. Successful inhibition was determined using fluorescent probes followed by microscopy, as described below.

### Sindbis Vector Infection

Sindbis vectors (SV-GFP and SV-Luc) were produced as described previously [6]. Briefly, plasmids carrying the replicon (SinRep-GFP or SinRep-Luc) or DHBB helper RNAs were linearized with PacI, NotI or XhoI respectively. *In vitro *transcription was performed using the mMessage mMachine RNA transcription kit (Ambion). Helper and replicon RNAs were then electroporated into BHK cells and incubated in αMEM supplemented with 10% FCS for 12 hours. After 12 hours the media was replaced with OPTI-MEM (GIBCO-BRL) supplemented with CaCl_2 _(100 mg/l) and cells were incubated at 37°C for 24 hours, at which time the supernatant was collected and frozen at -80°C. Vectors were titered as described previously [6]. The cells were infected with Sindbis viral vector as described previously [5] in OPTI-MEM + CaCl_2 _at a multiplicity of infection of 100, to achieve greater than 85% infectivity as assessed by FACS analysis. Briefly, cells were incubated with indicated vector for 1 hour at room temperature with gentle agitation. In parallel, cells were incubated in OPTI-MEM + CaCl_2 _(mock infected). Cultures were washed with PBS and incubated in complete media at 37°C for indicated times. For each sample, (except those used in microscopy experiments) expression of GFP was used to assess infectivity through the presence of the fluorescent protein, and replication was assessed by monitoring intensity by FACS analysis. Time post infection was calculated from the time the vector was first added to the cells at room temperature.

### FACS Analysis

To assess infectivity or transfection efficiency FACS analysis was employed. Briefly, cells were washed with PBS and incubated with trypsin-EDTA (Mediatech) for 5 minutes at 37°C. Cells were centrifuged at 300 × g for 5 minutes at 4°C, washed one time with PBS and centrifuged at 300 × g. Cells were resuspended in PBS. Cells were fixed by the addition of a 4% paraformaldehyde solution. Samples were run on a FACSCaliber instrument (Beckton Dickson) and data was analyzed using FlowJo version 8.2 software (Tree Star, Inc.).

### Short Interfering RNA Studies

For ablation of protein expression siGENOME SMARTpool siRNA directed against PKR, Bad, Bik or Bak was used. siGLO, a fluorescently labeled RISC-free siRNA was used as a negative control (Dharmacon). Briefly, each transfection was performed in a 12 well plate. Cells were plated to 70% confluency. Dharmafect reagent IV (Dharmacon) was incubated with each oligo to a final RNA concentration of 100 nmol/l. Then, 0.8 ml of antibiotic-free media was added to the mixture, which was then added to the MOSEC or Pan02 cells. After 24 hours, cells were plated according to downstream experiments. Use of siGLO enabled the calculation of transfection efficiency through FACS analysis. For each experiment only samples with greater than 80% transfection efficiency were used. Knockdown was confirmed by western blot and cells were used for experiments within the optimal knockdown time period.

### Transfection

MOSEC cells were transiently transfected with GADD34 expressed in a CMV2-based mammalian expression vector [55], GADD34 PP1c mutant protein cloned in a pBABE puro expression vector [55], both a generous gift from Dr. David Ron at New York University, or Mcl-1 expressed in a CMV6-based mammalian expression vector (Origene). Briefly, MOSEC cells were plated on 6 well plates. At 70% cell confluency, 2 ug of either GADD34, GADD34 PP1c mutant or Mcl-1 plasmid was transfected using Fugene reagent (Roche). Cells were incubated at 37°C for 24 hours at which time they were plated for downstream applications. In each experiment a control vector expressing GFP under a CMV promoter was used to assess transfection efficiency and control for the presence of exogenous DNA.

### Western Blotting

Cell lysates were collected using MPER (Pierce) supplemented with protease (Roche) and phosphatase (Pierce) inhibitors, according to manufacturer instructions. Samples containing 25 ug of total protein were run on polyacrylamide gels (BioRad) under reducing conditions. Protein was transferred to polyvinylidene fluoride membrane (Millipore). Membranes were probed with anti PKR (Santa Cruz), anti phospho-eIF2α (Cell Signaling Technologies), anti phospho-JNK (BD Bioscience), anti β-actin (Sigma), anti Mcl-1 (Santa Cruz), anti 14-3-3 pan (Millipore), anti Bak (Cell Signaling), anti Bad (Abcam), anti Bik (Cell Signaling) or anti phospho-threonine (Sigma) antibodies. Horseradish peroxidase conjugated secondary antibodies were used (Santa Cruz) and then samples were exposed with the SuperSignal West Pico Chemiluminescence substrate (Pierce) according to manufacturer instructions.

### Immunoprecipitation

MOSEC cells were collected and anti Bcl-xl and anti Mcl-1 samples were subjected to mitochondrial isolation by differential centrifugation with the Mitochondrial Isolation Kit for Cultured Cells (Pierce), according to manufacturer instructions. Dynal beads (Invitrogen) were incubated with 5 ug antibody (anti Bad (Cell Signaling), anti Bcl-xl (Cell Signaling), anti Mcl-1 (Santa Cruz) or PKR (Santa Cruz)), for one hour with agitation. Antibodies were cross-linked to beads using Dimethylpimelimidate (Pierce), for 45 minutes with agitation. Beads were then incubated with lysate overnight at 4°C, with agitation. Samples were washed with PBS with 0.05% Tween^® ^20 four times. Protein was eluted from beads with 0.1 M citric acid pH 2.7. Then 50 mM Tris pH 8.0 was added to the sample to neutralize the acidic pH. Samples were subjected to western blotting as described above.

### ^35^S Labeling

MOSEC cells were infected with Sindbis vector as described above. At 8 h.p.i. for PKR samples and 24 h.p.i. for GADD34 transfected, siRNA transfected and JNK inhibited samples, cells were labeled with ^35^S-methionine/cysteine (20 μCi/ml) (PerkinElmer) in methionine-free media for 2 hours. Unbound label was washed out and cells were incubated in DMEM supplemented with 4% FCS for 30 minutes. Lysates were collected with MPER (Pierce) and equal amounts of protein (20 μg) were run on a 4-20% gradient gel (BioRad). The gel was fixed with 50% Methanol 10% Acetic Acid for 30 minutes at room temperature. The gel was then incubated in enhancer solution (GE Healthcare) for 10 minutes and then dried for 2 hours at 80°C under vacuum. The gel was exposed to film overnight at -80°C.

### Cell Viability

Cells were cultured on 96 well luminescence plates (BD Bioscience). After initial treatments of siRNA transfection, DNA transfection or JNK inhibition cells were infected with SV-GFP. In parallel, a 6 well plate receiving the same treatment was harvested for FACS analysis to monitor infectivity. After 24 hours, cell viability was assayed with the Cell Titer Glo assay (Promega). Briefly, Cell Titer Glo reagent was added 1:1 directly to media. Samples were incubated for 2 minutes at room temperature with agitation and then incubated for an additional 10 minutes. After incubation luminescence was read in RLU with a multiwell plate reader, Wallac EnVision (Perkin Elmer). To calculate cell viability each sample was compared with the mock-infected sample of the same treatment and then corrected for percent infectivity, according to the formula below.

### Immunofluorescence

For TIA-1, eIF4E, eIF4G and Bax immunofluorescence, MOSEC cells were cultured on chamber slides (BD Bioscience). At 6 h.p.i. with SV-Luc, for TIA-1, eIF4E and eIF4G, and 20 h.p.i. for Bax, samples were processed for immunofluorescence. For Bax immunofluorescence a positive control of staurosporine treated cells was processed in parallel. Briefly, cells were washed with PBS, fixed with 4% paraformaldehyde and permeabilized with 1% Triton X100 as necessary. Cells were blocked in PBS containing 0.1% Triton X100 and 3% BSA. Slides were probed with anti TIA-1 (Santa Cruz), anti eIF4E (Santa Cruz), anti eIF4G (Santa Cruz) or anti Bax (Santa Cruz) overnight at 4°C. Slides were then washed and incubated with Alexafluor 488 and Alexafluor 594 secondary antibodies (Molecular Probes) and mounted with Prolong Gold Antifade Reagent (Invitrogen).

Caspase 3 reagent (Biotium), Caspase 8 and 9 reagents (Immunochemistry Technologies Inc.), were used for live cell imaging. For caspase 8 and 9 MOSEC cells were cultured on 12 well plates (BD Bioscience). For caspase 3 staining MOSEC cells were cultured on 35 mm glass bottom microwell dishes (MatTek Cultureware). At 24 h.p.i. with SV-Luc, cells were washed with PBS and caspase reagent was added. Cells were incubated for 30 minutes at 37°C. Samples were washed with PBS; Caspase 8 and caspase 9 samples were visualized with a Nikon Eclipse TE200-E microscope (Nikon Instruments Inc.) with a plan-fluor 10×/0.30 Ph1 DL objective lens. Images were captured with a digital camera (Cool Snap EZ, Photometrics) at room temperature using the NIS-Elements BR3.0 imaging software (Nikon). TIA-1, eIF4E, eIF4G, Bax and caspase 3 samples were visualized with a microscope (Axiovert 100 m; Carl Zeiss MicroImaging, Inc.) fitted with a plan-Apochroma 100/1.40 oil DIC objective lens. Images were captured with a digital camera (DKC-5000; Sony) at room temperature using the LSM510 version 3.2 SP2 program (Carl Zeiss MicroImaging, Inc.). The images were cropped to illustrate a representative field and RGB image capture was divided into individual channels for single color visualization with Adobe Photoshop 8.0.

## Abbreviations

(LAMR): 37/67 high affinity laminin receptor; (TUNEL): terminal deoxynucleotidyl transferase [TdT]-mediated dUTP nick end labeling; (PKR): double-stranded RNA activated protein kinase; (eIF2α): eukaryotic translation initiation factor 2 alpha, (JNK): c- Jun N-terminal kinase; (TNFα): tumor necrosis factor alpha; (Mcl-1): myeloid cell leukemia-1; (Bcl-2): B-cell lymphoma 2; (Bak): Bcl-2 homologous antagonist killer; (Bax): Bcl-2 associated X protein; (Bcl-xl): B-cell lymphoma extra large; (Bad): Bcl-2 associated death promoter; (Bik): Bcl-2 interacting killer; (MOSEC): mouse ovarian surface epithelial cells; (h.p.i.): hours post infection; (GADD34): Growth Arrest and DNA Damage-inducible protein 34; (PP1c): protein phosphatase 1c; (TIA-1): T-cell-restricted intracellular antigen; (eIF4E): eukaryotic translation initiation factor 4E; (eIF4G): eukaryotic translation initiation factor 4G; (IFN): interferon; (SFV): Semliki Forest Virus; (BHK): Baby hamster kidney; (αMEM): Minimum Essential Medium Eagle, Alpha Modification; (FCS): fetal calf serum; (DMEM): Dulbecco's Modification of Eagle Medium; (FACS): fluorescence activated cell sorting; (PBS): phosphate buffered saline; (MPER): mammalian protein extraction reagent; (RLU): relative luciferase units.

## Competing interests

Some of the authors have competing interests. Specifically, the contents of this paper are being utilized for a patent. According to the rules and regulations of New York University School of Medicine, if this patent is licensed by a third party, the authors (DM, LV) may receive benefits in the form of royalties or equity participation.

## Authors' contributions

LV conceived of the experiments, carried them out and drafted the manuscript. DM helped with experimental design, provided funding and input on the manuscript. LV and DM have read and approved the final manuscript.
